# Supplementary material for: Content-rich biological network constructed by mining PubMed abstracts
Source: BMC Bioinformatics. 2004 Oct 8;5:147. doi: 10.1186/1471-2105-5-147 (PMC528731; doi:10.1186/1471-2105-5-147)
Supplement: Additional File 2 — The original results of the above study (non-essential files are deleted to keep the file size under the limit set by BMC bioinformatics). [file 1471-2105-5-147-S2.bz2 › chilibotAdditionalFile2/dip05/623ID9103205E11009/html/GRPE_DNAK.html]

 


 **GRPE** and **DNAK** 
  
Found 298 abstracts in PubMed, retrieved 05.  
 

 What does Google say? 
 PDF only 
| .edu only 

---

**Interactive relationship** (e.g. stimulation, inhibition, etc)

**Neutral relationship**- This information is important for understanding the conformational changes  **GrpE**  undergoes on binding to  **DnaK** .  Ref: 12840016 J Biol Chem< MedlineTA>J Biol Chem,
- We show that the equilibrium dissociation constants for the interaction of  **GrpE**  with wtDnaK, lidless  **DnaK**  2 517, the ATPase domain 2 388, and the substrate binding fragment 393 507 are 64 16 nM, 4.0 1.5 nM, 35 10 nM, and 67 11 μM, respectively,.  Ref: 12885236 BiochemistryBiochemistry,
- Such results strongly suggest that the 33 residue flexible N terminal tail of  **GrpE**  binds in the substrate binding pocket of  **DnaK** .  Ref: 12885236 BiochemistryBiochemistry,
- Our results reveal a complex set of molecular interactions between  **DnaK**  and its cochaperone  **GrpE** .  Ref: 12885236 BiochemistryBiochemistry,
- The co chaperone  **GrpE**  is essential for the activities of the Hsp70  [ **DNAK** ]  system which assists protein folding.  Ref: 12840016 J Biol Chem< MedlineTA>J Biol Chem,
- Deletion of  **DnaK**  s Lid Strengthens Binding to the Nucleotide Exchange Factor,  **GrpE**  A Kinetic and Thermodynamic Analysis.  Ref: 12885236 BiochemistryBiochemistry,
- **GrpE**  is the nucleotide exchange factor for the Escherichia coli molecular chaperone  **DnaK** , the bacterial homologue of Hsp70  [ **DNAK** ] .  Ref: 12885238 Biochemistry, 2003
- In turn, it was proposed that temperature dependent down regulation of the activity of  **GrpE**  may increase the time in which  **DnaK**  binds its substrates at higher temperatures.  Ref: 12885238 Biochemistry, 2003

**Non-interactive relationship** (e.g. studied together, co-existance, homology, etc.)

- Experiments were geared toward understanding the influence of  **DnaK**  s three domains, the ATPase residues 1 388, substrate binding residues 393 507, and lid residues 508 638 domains, on complex formation with  **GrpE** .  Ref: 12885236 BiochemistryBiochemistry, 2003
- Active protein disaggregation by a chaperone network composed of ClpB and  **DnaK**  DnaJ  **GrpE**  is essential for the recovery of stress induced protein aggregates in vitro and in Escherichia coli cells.  Ref: 12828638 Mol Microbiol< MedlineTA>Mol Microbiol, 2003
- The possibility that deletion of  **DnaK**  s lid creates a more symmetrically shaped molecule, with enhanced affinity to  **GrpE** , is also discussed.  Ref: 12885236 BiochemistryBiochemistry, 2003
- However, in these conditions, the Escherichia coli  **GrpE**  dimer is asymmetric since the monomer that binds  **DnaK**  adopts an open conformation.  Ref: 12840016 J Biol Chem< MedlineTA>J Biol Chem, 2003
- In the presence of excess nickel, NikR activated the transcription of ureA ureB hp72 73, nixA hp1077, copA2 hp1072, hpn hp1427 and hpn like hp1432 genes and repressed the expression of genes encoding proteins involved in ferric iron uptake and storage pfr hp0653, fur hp1027, frpB4 hp1512, exbB exbD hp1339 1340, ceuE hp1561, motility cheV hp616, flaA hp0601, flaB hp0115, stress responses hrcA  **grpE**   **dnaK**  hp111 110 109 and encoding outer membrane proteins omp11 hp0472, omp31 hp1469, omp32 hp1501.  Ref: 12890020 Mol Microbiol, 2003
- SPR experiments revealed that  **GrpE**   **DnaK**  393 507 complex formation is inhibited by added peptide and abolished when the 33 residue flexible tail of  **GrpE**  is deleted.  Ref: 12885236 BiochemistryBiochemistry, 2003
- In this study, we have used surface plasmon resonance SPR and isothermal microtitration calorimetry ITC to study the mechanism of complex formation between the Hsp70  [ **DNAK** ]  molecular chaperone,  **DnaK** , and its cochaperone,  **GrpE** , which is a nucleotide exchange factor.  Ref: 12885236 BiochemistryBiochemistry, 2003
- The low resolution structure of human  **GrpE**  presented here suggests that  **GrpE**  is a symmetric dimer when not bound to  **DnaK** .  Ref: 12840016 J Biol Chem< MedlineTA>J Biol Chem, 2003
- Small angle X ray scattering analysis shows the protein possesses an elongated shape in solution, and demonstrates that its envelope, determined by an ab initio method, is similar to the high resolution envelope of Escherichia coli  **GrpE**  bound to  **DnaK**  obtained from single crystal X ray diffraction.  Ref: 12840016 J Biol Chem< MedlineTA>J Biol Chem, 2003
- It is of considerable importance for structural  **GrpE**  research to answer the question of whether the  **GrpE**  dimer is only asymmetric while bound to  **DnaK**  or also as free dimer in solution.  Ref: 12840016 J Biol Chem< MedlineTA>J Biol Chem, 2003
- This unique mode of binding between  **GrpE**  s tail and  **DnaK**  contributes to, but does not fully explain, the decrease in K d from 64 to 4 nM upon deletion of  **DnaK**  s lid.  Ref: 12885236 BiochemistryBiochemistry, 2003
